# Improving practice in community-based settings: a randomized trial of supervision – study protocol

**DOI:** 10.1186/1748-5908-8-89

**Published:** 2013-08-10

**Authors:** Shannon Dorsey, Michael D Pullmann, Esther Deblinger, Lucy Berliner, Suzanne E Kerns, Kelly Thompson, Jürgen Unützer, John R Weisz, Ann F Garland

**Affiliations:** 1 Department of Psychology, University of Washington, 335 Guthrie Hall, Seattle, WA 98195, USA; 2 Psychiatry and Behavioral Sciences, University of Washington School of Medicine, 2815 Eastlake Avenue East, Suite 200, Seattle, WA 98102, USA; 3 School of Osteopathic Medicine, Rowan University, 42 E. Laurel Road, Suite 1100, Stratford, NJ 08084, USA; 4 Harborview Center for Sexual Assault and Traumatic Stress, 401 Broadway, Suite 2027, Seattle, WA 98122, USA; 5 Psychiatry and Behavioral Sciences, University of Washington School of Medicine, 1959 Northeast Pacific Street, Room BB-1661A, Seattle, WA 98195, USA; 6 Department of Psychology, Harvard University Medical School, 1030 William James Hall, 33 Kirkland Street, Cambridge, MA 02138, USA; 7 Department of School, Family, and Mental Health Professions, School of Leadership & Education Sciences, University of San Diego, 5998 Alcalá Park, San Diego, CA 92110, USA

**Keywords:** Evidence-based treatment, EBT, Supervision, Training, Implementation, Fidelity, Behavioral rehearsal, Symptom monitoring, Role-play, Trauma-focused cognitive behavioral therapy

## Abstract

**Background:**

Evidence-based treatments for child mental health problems are not consistently available in public mental health settings. Expanding availability requires workforce training. However, research has demonstrated that training alone is not sufficient for changing provider behavior, suggesting that ongoing intervention-specific supervision or consultation is required. Supervision is notably under-investigated, particularly as provided in public mental health. The degree to which supervision in this setting includes ‘gold standard’ supervision elements from efficacy trials (e.g., session review, model fidelity, outcome monitoring, skill-building) is unknown. The current federally-funded investigation leverages the Washington State Trauma-focused Cognitive Behavioral Therapy Initiative to describe usual supervision practices and test the impact of systematic implementation of gold standard supervision strategies on treatment fidelity and clinical outcomes.

**Methods/Design:**

The study has two phases. We will conduct an initial descriptive study (Phase I) of supervision practices within public mental health in Washington State followed by a randomized controlled trial of gold standard supervision strategies (Phase II), with randomization at the clinician level (i.e., supervisors provide both conditions). Study participants will be 35 supervisors and 130 clinicians in community mental health centers. We will enroll one child per clinician in Phase I (N = 130) and three children per clinician in Phase II (N = 390). We use a multi-level mixed within- and between-subjects longitudinal design. Audio recordings of supervision and therapy sessions will be collected and coded throughout both phases. Child outcome data will be collected at the beginning of treatment and at three and six months into treatment.

**Discussion:**

This study will provide insight into how supervisors can optimally support clinicians delivering evidence-based treatments. Phase I will provide descriptive information, currently unavailable in the literature, about commonly used supervision strategies in community mental health. The Phase II randomized controlled trial of gold standard supervision strategies is, to our knowledge, the first experimental study of gold standard supervision strategies in community mental health and will yield needed information about how to leverage supervision to improve clinician fidelity and client outcomes.

**Trial registration:**

ClinicalTrials.gov NCT01800266

## Background

A substantial number of evidence-based treatments (EBTs) for child and adolescent mental health disorders exist, many with multiple randomized controlled trials (RCTs) supporting efficacy in improving outcomes and functioning [1]. EBTs are treatment approaches that have met strict criteria for efficacy based on rigorously controlled studies [2]. However, outcomes for youth served in community-based, public mental health settings have been less positive [3], and direct randomized comparisons of EBTs to usual treatment in such settings have shown variable findings and modest mean effect sizes [4,5]. Initial efforts to bridge the gap by providing access to EBTs through clinician training were mostly insufficient, as they did not include a focus on key implementation factors beyond training, and resulted in little to no change in clinician practice [6]. Therefore, EBT implementation in community-based settings has been viewed as proceeding at ‘an unacceptably slow pace’ [7]. Training in an EBT may be necessary—but not sufficient—to improve client outcomes for the average client served. Two recent reviews of clinician training concluded that training efforts must be active, multi-component, and attend to contextual factors such as organizational support [8,9]. One organizational support factor highlighted as critical, yet receives little research attention, is supervision. According to Herschell [9], the field requires ‘a better understanding of how supervisors should be trained and included in the implementation process.’ Clinical supervision and consultation are potentially the ‘least investigated’ aspects of implementation [10,11] despite findings that supervision accounts for a significant proportion of variance in client outcomes (16%) [12].

### The role of community-based supervisors in supporting EBT delivery

The majority of community-based mental health settings provide some form of ongoing clinical supervision [13], yet the role of community-based supervisors (CBS) in supporting EBT has been largely overlooked [14]. Local supervision, unlike external expert consultation, is generally included within existing organizational and funding structures. Community-based supervision holds promise as a more natural and potentially sustainable mechanism for supporting EBT if used effectively. Very little research—most employing non-experimental designs—has focused on CBS supervision strategies, such as content, techniques (e.g., active listening, role-play, review of case notes) and focus (e.g., past sessions, upcoming sessions), and how CBS can feasibly and effectively support clinicians in providing EBT [15,16]. This has created a critical gap in the implementation literature. What is known suggests that supervision varies considerably and rarely, or inconsistently includes ‘gold standard’ supervision strategies, with limited attention to common EBT techniques like assignment and review of client homework [17,18]. CBS have a variety of competing supervision demands, including overseeing billing, productivity, case management, professional development, and administrative tasks—all important from the organization’s perspective—as well as supervising actual clinical content and interventions. It may be challenging for CBS to allocate sufficient time to the types of supervision activities that are commonly part of supervision in RCTs, and may be necessary to support EBT, such as ongoing skill building through practice and feedback and observation of actual practice [19,20], when balancing all of these responsibilities. Additionally, the dose of supervision may be smaller in community settings than in RCTs. When looking within the broader supervision literature (i.e., beyond CBS), not only are experimental studies missing, but available studies are plagued by a variety of methodological flaws, including small sample sizes, reliance on only self-report, and problematic outcome measures [10].

Because clinicians are the direct service providers, most dissemination and implementation (DI) efforts predominantly or exclusively focus on clinicians, despite high turnover rates. In comparison, supervisors, although providing less direct service, have significant influence on clinicians and may have lower rates of turnover (e.g., [21]). To facilitate DI, Chorpita and Regan [22] suggest that we may need to advance beyond examining practices with real-world clinicians and clients to examining ‘real-world supervisors and managers.’ In particular, ongoing supervision support for providers may be even more important than initial training. In a recent RCT of varying training approaches, the dose of model-specific supervision received, and not the training approach, predicted clinician fidelity to cognitive behavioral therapy (CBT) practice post-training [23].

### ‘Gold standard’ supervision strategies

In efficacy trials and expert consultation models, a relatively common set of ‘gold standard’ supervision strategies have been used to support clinician fidelity and, in turn, positive client outcomes [24-27]. Fidelity is defined as ‘the degree to which an intervention was implemented as it was prescribed in the original protocol as it was intended by the program developers’ [28]. These supervision strategies typically include some combination of four elements: fidelity monitoring, skill building/behavioral rehearsal, review of actual practice (via live observation or tape review), and symptom monitoring. However, there is little empirical guidance around which strategies are most critical, which combinations are most effective, and how these elements can be implemented effectively and efficiently (i.e., feasibly) in community-based settings [29]. According to Garland et al. [30], defining ‘what works’ in implementation research must also include what is ‘practical, feasible, and affordable, and therefore, what is effective.’

Each of these four ‘gold standard’ supervision elements can be challenging to implement in community-based care given resource limitations [31]. This is certainly true of fidelity or treatment integrity monitoring, as priorities of valid, effective measurement must be balanced by priorities for efficient measurement (e.g., feasible, acceptable) [26]. For example, indirect methods such as clinician self-report have relatively poor concordance with objective observer ratings of actual in-session practice (e.g., [32]), but direct methods, including review of actual in session practice, are more expensive and time-consuming [33,34] and ‘differ considerably from most psychotherapy supervision’ [16]. Alternatively, skill building/behavioral rehearsal (BR) may provide a particularly cost-effective proxy for obtaining objective ratings of actual practice in community settings [35], in that the supervisor can observe demonstration of core treatment elements via role-play [36,37]. When combined with other strategies, BR may be a valid, feasible, and effective fidelity monitoring strategy that also allows for ongoing skill building and coaching to fidelity [36,38]. Of note, BR with simulated patients is considered a form of direct observation [33,39] in medical education, but it has received little research attention in mental health.

In contrast to the above clinician-focused gold standard strategies, symptom monitoring (SM) is a different, client-focused gold standard supervision element used in many efficacy trials and is a primary element in measurement-based practice [40]. Ongoing client SM provides a means for regularly assessing client functioning and response to the intervention [41], as it allows the supervisor (and clinician) to monitor client response even in the absence of direct client observation [42,43]. Like the other gold standard strategies, SM has not been fully implemented in most community settings [44,45]. Ideally, supervision integrates data on clinician practice (first three gold standard strategies) with data on client response (SM), to highlight when and to what extent intervention approaches are effective or ineffective.

### Supervision, fidelity, and client outcomes

One of the primary goals of the gold standard supervision strategies is to improve clinician fidelity, as the research literature generally supports a link between model fidelity and client outcomes (e.g., [46,47]; see [48] for an exception) although the strength of the relation varies. Stronger associations between fidelity and outcomes are seen in effectiveness trials, likely due to required high fidelity in efficacy trials creating a floor effect [49]. The literature on the association between supervision and client outcomes is limited but important, as improved client outcomes are the ‘acid test’ for defining good supervision [50]. Available studies suggest that supervision focused on coaching to fidelity predicts better client outcomes [16,51], which may be particularly important when providers are confronted with more heterogeneous or challenging clients [52]. There is also evidence that supervision is related to broader implementation outcomes beyond fidelity. Specifically, EBT coaching in supervision [53] and quality of supervision [54] have been associated with decreased burnout or emotional exhaustion, turnover intention [54], and actual turnover among clinicians [53].

To our knowledge, studies experimentally testing gold standard supervision strategies in community settings have not been conducted, limiting knowledge about how to leverage an important existing mechanism in community mental health to support EBT delivery. Studies are needed that describe usual care supervision, moving beyond self-report, and most importantly, that empirically test the differential impact of implementing gold standard supervision strategies in usual care. To maximize the impact of implementation efforts in the area of supervision, the field needs practical recommendations taking efficacy, efficiency, and feasibility into account to address questions such as: What are the current supervision strategies most commonly utilized by CBS trained in EBT? What techniques do they use, what content (e.g., EBT components, case management) is discussed, and temporally, on what do they predominantly focus (past sessions; upcoming sessions)? Which gold standard supervision strategies, if incorporated into CBS supervision, predict better clinician fidelity and client outcomes?

## Methods/Design

We propose a two-phase, mixed within- and between-subjects design studying supervision of an EBT for child trauma exposure sequelae, Trauma-focused Cognitive Behavioral Therapy (TF-CBT) [55] (see [1] for a review). In Phase I: Baseline and Descriptive Study, we will examine current ‘usual care’ supervision strategies (i.e., supervision baseline) of CBS who are trained in and supervise TF-CBT—including content, techniques, and focus, particularly the use of any gold standard techniques. Phase I will also yield some information on supervision dose (e.g., duration per clinician, per case; weekly consistency or inconsistency). In Phase II: Randomized Trial, clinicians will be randomized to one of two supervision conditions, both of which systematically include gold standard strategies: Symptom and Fidelity Monitoring (SFM) or SFM plus Behavioral Rehearsal (SFM + BR). In SFM, the focus is on systematic monitoring of client symptoms and fidelity, but only via clinician self-report given the challenges of direct methods (e.g., observation of actual practice) in community-based settings. In SFM + BR, the addition of BR provides a strategy that is potentially both a feasible proxy for direct methods and an opportunity for skill building. Primary aims across both phases are to describe current supervision practices and to examine the impact of including gold standard elements on clinician fidelity and client outcomes.

Our design leverages and builds on the existing funding and efforts provided through the Washington State Trauma-focused Cognitive Behavioral Therapy Initiative (WA TF-CBT Initiative). The structure for the WA TF-CBT Initiative is similar to the other 17 statewide initiatives (see [56-58]) and to national and statewide DI efforts of other EBT (e.g., NY’s Achieving the Promise for Children, Youth, and Families [59]). Since 2007, the initiative has trained clinicians and supervisors from across the state in TF-CBT. It provides some supervisor-specific training (one-day training yearly) and support (i.e., monthly consultation call, listserv) for CBS. The proposed study builds on this initiative, offering a unique opportunity to study supervision of an EBT in community-based settings. Participation in the state-funded WA TF-CBT Initiative has been geographically diverse, with involvement from over 60 public mental health agencies and nearly 900 clinicians and CBS statewide. These agencies are from urban and rural areas, some of which serve predominantly ethnically diverse populations.

### Clinician and supervisor participants

Participants will include 35 supervisors and 130 clinicians from an estimated 15 to 20 agencies. Agencies were selected from those in the WA TF-CBT Initiative with consideration of statewide geographic representation. Each agency has one to four supervisors and a potential clinician participant pool of 2 to 15 qualifying clinicians based on study inclusion and exclusion criteria. Eligible supervisors were identified during study development with additional supervisors recruited at the beginning of Phase I. Eligible clinicians must be: trained in TF-CBT as part of the WA TF-CBT Initiative and/or have completed the TF-CBT web training with at least one TF-CBT case underway; at least 50% FTE employees at one of the participating agencies; supervised by one of the participating supervisors; and able to provide TF-CBT in English. We propose only two exclusionary criteria: immediate plans to leave the agency or transition into a non-child/adolescent caseload carrying position. Clinicians and supervisors who exit the study will be replaced with new clinicians and/or supervisors who are similarly trained in TF-CBT and study protocols.

### Children and adolescents

We will recruit one child/family per clinician in Phase I, and three children/families per clinician in Phase II. Eligible children and adolescents will be clients at one of the participating agencies. Inclusion criteria include: age 6–17; trauma history; significant posttraumatic stress (PTS) symptoms; live with a parent/legal guardian who is willing to participate in the study; the child is English-speaking; and treatment approach will be TF-CBT. Exclusionary criteria are intentionally limited and include only: presence of a pervasive developmental disorder or cognitive impairment and parental serious mental illness. Clinicians are asked to introduce the study to caregivers of all potentially eligible youth who meet these inclusion and exclusion criteria. If interested, study staff proceed with recruitment.

### Children and adolescent data collection

Youth and parent measures will be collected by study staff via telephone at the beginning of treatment, and at three and six months into treatment (approximating end of treatment; post-treatment data for some and ‘still in treatment’ for others).

### Phase I study

Phase I will involve describing current supervision practices and establishing a baseline for current practice, specifically identifying extent of use of gold standard strategies and examining any association with clinician fidelity and client outcomes.

### Procedure

Clinicians and supervisors will complete self-report study measures via a web-based survey at the beginning and the end of Phase I. At the beginning of Phase I, clinicians and supervisors will participate in a two-day study procedures and TF-CBT booster training. Study training will focus on youth recruitment and mobile tablet device training (e.g., session audio recording, uploading, and data collection from supervisors/clinicians). Supervision meetings with study clinicians will be audio recorded using tablet devices and electronically submitted to our research team using a secure Health Insurance Portability and Accountability Act (HIPAA)-compliant server. In Phase I, 15 randomly selected meetings per supervisor will be coded, with selection distributed across participating clinicians (approximately 400 meetings). A random sample of three TF-CBT sessions per clinician, per study client, will be coded for TF-CBT fidelity. All coders will be trained to an established criterion of 80% agreement on training tapes. During the study, a random 20% of all coded sessions (supervision and TF-CBT) will be coded by two coders to test inter-rater reliability. If reliability is less than 80% on two successive reviews, booster training will be conducted.

### Measures

The primary outcomes of interest are supervision practices, clinician fidelity, and client outcomes. We also examine factors that may relate to supervision practices and fidelity, including supervisor and clinician background, training and experience, therapist knowledge of TF-CBT practice, therapist-supervisor relationship, organizational climate, attitudes towards EBTs, and therapist burnout.

### Objective supervision practices

No existing coding measure was identified that captures general and EBT (e.g., TF-CBT) strategies, including temporal focus (i.e., focus on past sessions vs. planning for future sessions). Items on the supervision coding measure for the proposed study were identified by review and synthesis of: an existing, psychometrically valid CBT-focused coding measure (i.e., Supervision: Adherence & Guidance Evaluation; D. Milne, PhD & R. Reiser, PhD, unpublished measure, 2008); a review of supervision interventions [60]; and a self-report supervision questionnaire used in Accurso’s CBS pilot study [17]. Similar to the Garland et al. [30] revised Therapy Process Observational Coding System for Child Psychotherapy (PRAC TPOCS-S), which itself is an adapted version of the original TPOCS-S [61], our supervision coding measure includes codes for supervision content (e.g., TF-CBT components; administrative aspects; assignment/review of client homework) and supervision techniques (e.g., symptom review; case note review; behavioral rehearsal), as well as extensiveness (e.g., frequency of use/time spent; thoroughness; continuous variable: 0 to 6) of content and techniques. We will also code temporal focus of supervision—relative percent of supervision focused on past sessions compared with planning for future sessions.

We will collect two self-report measures of supervision. Both supervisors and clinicians will complete: a revised version of the Supervision Process Questionnaire (SPQ) [17]; questions on frequency and type of supervision received; and questions on TF-CBT-specific supervision from two unpublished measures: the Project BEST (National Crime Victims Research and Treatment Center MUSC, 2010) and the Project TF-CBT New Jersey Pre-Work Surveys (PWS) (Child Abuse Research Education and Service Institute, Rowan University 2013). The SPQ collects estimates of supervision session time dedicated to ten supervision areas (e.g., crisis assessment, administrative tasks, case conceptualization, etc.). A small preliminary study has found fair to adequate inter-rater reliability for ratings of supervision areas for the original measure [17]. Published psychometric properties for items from the two unpublished measures) do not currently exist, but analyses will be conducted as part of this study.

### Objective TF-CBT fidelity

Audio recorded TF-CBT sessions will be coded using a TF-CBT specific version of the TPOCS developed by the study team, which builds on the TF-CBT Checklist Scoring Sheet (E. Deblinger, PhD and colleagues, unpublished measure, 2005) and Garland’s PRAC TPOCS-S [30]. Coding involves identifying prescribed TF-CBT content (e.g., psychoeducation, trauma narrative) and techniques (e.g., role-play, homework assignment/review) in child only, parent only, and conjoint parent–child sessions, as well as extensiveness of TF-CBT components and techniques, also with an extensiveness rating (0 to 6). Three randomly selected tapes per case, representing beginning, middle and end of treatment will be coded for fidelity, creating a continuous fidelity average for each case, for each clinician. For clinician-level fidelity analyses, fidelity across cases will be averaged by clinician.

### Child symptoms and functioning

To assess child PTS, study staff will administer the UCLA Post Traumatic Stress Disorder Reaction Index (PTSD-RI) [62], using a severity cutoff of 21 and higher or algorithm scoring for likelihood to meet diagnostic criteria. This 20-item measure assesses PTS and demonstrates good convergent validity and good to excellent test-retest reliability, with Cronbach’s α in the range of 0.90 for the total scale [63]. To assess child functioning, the Strengths and Difficulties Questionnaire (SDQ) will be administered, a 25-item measure with scales for Emotional Symptoms, Conduct Problems, Hyperactivity/Inattention, Peer Relationship Problems, and Prosocial Behavior [64]. Parents will complete the SDQ on all children; children 10.5 years and older will complete the self-report version of the SDQ. The SDQ has been found to have good discriminant validity, acceptable levels of test-retest reliability, and a Cronbach’s α of 0.73.

### Therapist/supervisor characteristics

We will use a study-created measure based on the Therapist Background Questionnaire [1] and the PWS to capture demographics, training, past experience, tenure in current organization, role, years in current role, caseload composition and size, other supervision received, and utilization of TF-CBT, among other important background characteristics.

### Supervisor-clinician relationship

Given the potential importance of the supervisor-clinician relationship, we will use three measures to assess this relationship: Leader Member Exchange [65], Supervisor Working Alliance Inventory (SWAI) [66,67], and the Supervision Alliance Scale (SAS) [66,68]. In Phase I, both supervisors and clinicians will report on the Leader-Member Exchange; only clinicians will report on the SWAI and SAS.

### Associated factors

We expect that a number of factors will be related to supervision, fidelity, and client outcomes, and therefore should be measured in both phases. Clinicians and supervisors will report on: their perceptions of their organization’s climate, resources, and clinician stress and autonomy using select subscales from the Texas Christian University Organizational Readiness for Change (TCU-ORC) [69]; their own TF-CBT Self Efficacy (from the PWS and a WA TF-CBT Initiative measure; S.D. and colleagues, unpublished measure, 2013) and supervisor self-efficacy in providing supervision (from the PWS); TF-CBT knowledge using multiple choice questions developed by our team and from the Denver Post Health Survey (M. Fitzgerald, PhD, unpublished measure, 2010); attitudes towards evidence-based practices using the Modified Evidence-Based Practice Attitude Scale (EBPAS) [70]; therapist burnout with the Maslach Burnout Inventory (MBI) Emotional Exhaustion Subscale [71]; and turnover intention using four items adapted from [72] and used in [54], with a Cronbach’s α of 0.85.

### Aim 1: Describe and establish baseline use of supervision strategies, when provided with implementation support

#### Analyses

We will analyze coded supervision sessions and self-report measures using grand means and variability (i.e., over all sessions) and supervisor-level means and variability for frequency and intensity, building on procedures in Garland et al. [30]. The SPQ and PWS will be analyzed with descriptives at baseline and we will explore for significant changes over time. These analyses will establish a baseline for the types of supervision practices used overall and within-supervisor variability. We will use the coded TF-CBT sessions to obtain a baseline for TF-CBT fidelity. We will explore univariate descriptives and change over time for all other measures described above, as well as the relation between client outcomes, clinician fidelity, and supervision content, strategies, and focus.

### Phase II study

In Phase II of the study, all clinicians will be randomized to one of two supervision conditions: Symptom and Fidelity Monitoring (SFM) or SFM plus Behavioral Rehearsal (SFM + BR). We will randomize by clinicians because they are our primary unit of analysis. Supervisors will provide both conditions to balance the impact of individual supervisor/agency factors across conditions. We will monitor supervisor adherence to condition protocol and experimental drift through coding of audiotapes of supervision sessions. During Phase II, four randomly selected meetings per supervisor (two for each condition, distributed across participating clinicians) will be reviewed within a two-week window of the supervision meeting occurring (which is possible due to near real-time uploading with the use of the mobile device system) by research assistants blind to study condition. If experimental drift is identified, supervisors will be contacted within two weeks for a booster training on the supervision conditions. Supervisors will receive a biweekly email on condition adherence so that all supervisors receive regular communication from our team related to provision of supervision conditions.

Phase II has the added benefit of a within-subject design, in which clinicians retained from Phase I serve as their own controls prior to randomization. Given clinician attrition and replacement, some clinicians will not have Phase I data, and therefore will only be part of the between-subject analyses. Fidelity scores will be obtained from the three Phase II enrolled clients per clinician to obtain precise and reliable estimates of effect (see earlier ‘Objective TF-CBT fidelity’ section) and to increase statistical power for detecting differences between the two intervention conditions.

### Description of phase II supervision conditions

#### Symptom and fidelity monitoring (SFM)

Symptom Monitoring involves clinicians monitoring key symptoms each session using brief measures via the tablet device. We use the measures recommended as part of the WA TF-CBT Initiative: the child-completed Screen for Child Anxiety-Related Emotional Disorders (SCARED)—Anxiety and PTS Subscales [73,74] to assess PTS/anxiety and the caregiver-completed externalizing subscale of the Pediatric Symptom Checklist-17 (PSC-17) [75] to assess externalizing behavior. Using a program created for this study, which builds on Dr. Unützer’s monitoring system [43], symptoms will be scored and graphed over time to provide a pictorial representation of improvement or deterioration, and reviewed in supervision. For Fidelity Monitoring, clinicians will complete a brief, standard TF-CBT checklist after each session (via tablet) that also will be reviewed in supervision. The supervisor will have a slightly elaborated version that includes follow-up queries for TF-CBT components and key CBT techniques (assigning/reviewing homework, role-play), and a cue to plan for the upcoming session. The goal is to determine if systematic monitoring and review—of symptoms and indirect fidelity—is sufficient to improve fidelity and client outcomes.

### Symptom and fidelity monitoring plus behavioral rehearsal (SFM + BR)

The SFM + BR condition includes the SFM components and an additional, multipurpose strategy—behavioral rehearsal (BR). Given concerns about relying on clinician self-report of fidelity (i.e., indirect methods) and the importance of skill building, the inclusion of BR is a potentially high-yield addition [27]. In SFM + BR, supervisors will conduct a TF-CBT component BR (e.g., five to ten minutes) relevant to an upcoming session in each supervision meeting and provide feedback. Supervisors will have a short set of guidelines (e.g., bulleted form) that detail expected content and techniques for BR of each TF-CBT component. Guidelines focus specifically on common challenges for TF-CBT components (e.g., dealing with avoidance, homework assignment/review).

### Procedure

In the beginning of Phase II, supervisors will participate in a two-day training on the Phase II supervision conditions and study procedures, and specifics on how to systematically integrate gold standard elements into supervision. Training will include didactic information, benefits of SFM and BR, modeling of SFM and SFM + BR use, and coached supervisor practice of SFM and SFM + BR procedures and their use in supervision meetings. Trainers will provide clinician vignettes and pre-loaded SFM data on the tablets for supervisors to use in practicing both experimental conditions. Following recommendations on using BR in training [36], the SFM + BR practice vignettes will also include a supervisor role-play guide for the clinician role, to make the role-plays and coached practice opportunities as helpful as possible. To reinforce their future use, supervisors will be asked to discuss how they think the strategies could enhance their supervision of TF-CBT. Training will also include orientation to the experimental design and trial procedures, with particular emphasis on the importance of condition adherence and preventing experimental drift.

### Clinician self-report TF-CBT fidelity

The Brief Practice Checklist (BPC; E. Deblinger, PhD and colleagues, unpublished measure, 2008), a clinician self-report checklist of TF-CBT components will be used in Phase II as the indirect fidelity monitoring strategy.

### Supervisor report of TF-CBT fidelity

A modified version of the BPC created by the study team, the Supervisor BPC (S-BPC), will be used to assess supervisor report of clinician fidelity to TF-CBT from supervision meetings.

### Acceptability and feasibility of the phase II conditions

The Supervisor Interview (SI) [76] is a qualitative interview and was used previously by our team to evaluate feasibility and acceptability of an expert consultation model for child welfare workers. The SI has been revised for this study to include questions specific to Phase II SFM and SFM + BR.

### Aim 2: Evaluate effects of supervision condition (SFM vs. SFM + BR) on clinician fidelity and client outcomes (phase II)

#### Analyses

We will use Hierarchical Generalized Linear Models (HGLM) to test Aim 2, with the hypotheses that fidelity and client outcomes will be higher for SFM + BR than SFM and higher for SFM than supervision in Phase I. A two-level HGLM will predict fidelity using individual-mean fidelity scores for each client per clinician as the dependent variable and supervision as the independent variable for client functioning. These analyses are sufficiently powered at 0.8 to detect small effect sizes (0.35) with clinician-level Intraclass Correlations Coefficients (ICCs) less than 0.04 and small to moderate effect sizes (0.4) with ICCs as high as 0.21. To test for client functioning, we will build three-level longitudinal models, with level one variables representing time and child functioning scores, level two representing child-level factors, and level three representing clinician-related factors. These analyses are sufficiently powered at 0.8 to detect small effect sizes with clinician ICCs up to 0.24 and all conceivable moderate or large effects.

### Aim 3: Test fidelity as a mediator of the relationship between supervision condition and client outcomes

#### Analyses

Mediational analyses will employ HGLMs using methods that extend traditional mediational modeling [77] to a multi-level longitudinal framework [78-80].

### Aim 4: Explore the relationship between supervision condition and broader implementation factors, including feasibility, acceptability, and impact on agency-relevant outcomes

#### Analyses

We will perform separate analyses on the dependent variables of turnover intention, actual turnover, burnout, supervision alliance, attitudes towards EBTs, and organizational climate. Mixed between- and within-subjects ANOVAs will test for differences between supervision conditions (between-clinician) and Phase I and Phase II (within-clinician) differences while controlling for important covariates. To examine feasibility and acceptability, we will use qualitative interview methods. Interviews will be transcribed and coded in the NVivo software program [81] using an integrated grounded theory approach to capture identified categories of interest as well as subcategories or emergent categories. Thematic content analysis, using the constant comparative method, also will be conducted [82,83].

#### Trial status

The Washington State Institutional Review Board has approved the study procedures. We have already enrolled supervisors and clinicians and begun Phase I data collection. At the time of submission of this manuscript (May 2013), we are continuing recruitment for parent–child dyads and Phase I data collection. Phase II will begin in September 2013.

## Discussion

### Innovation and potential impact

The proposed project is innovative at three levels. First, it addresses a critical gap in the implementation literature by focusing on potential strategies for improving supervision with the primary goal of enhancing clinician delivery of EBTs in community-based settings.

Second, we plan to accomplish two goals in a single trial [84]. We will describe ‘usual care’ supervision for CBS trained in EBT, including EBT content and strategies, which to date has not been done. We also will test practical and feasible gold standard supervision strategies that, if found effective, will have significant public health impact (Strategic Objective 4 [85]). The ‘gold standard’ or experimental supervision strategies were specifically selected for potential effectiveness, feasibility, and potential for scale-up in community-based settings. This two-phased design will yield findings early in the trial about existing strategies and focus of supervision and, towards the end of the trial, will yield information about the impact of supervision enhancements to support EBT (i.e., systematic use of gold standard strategies) on clinician fidelity and client outcomes. A specific innovation is that one of the strategies tested is the addition of BR, which may have less context validity than observation, but potentially is a more feasible, multipurpose strategy for both assessing fidelity (via proxy) and improving skills [36,38].

Third, the study uses a relatively low-cost mobile platform that holds promise for greater use of technology in the diverse settings in which mental healthcare is increasingly provided (e.g., in-home, school, residential settings). According to Pringle et al. [84], ‘implementation studies too often neglect the potential contributions of emerging technologies to help integrate effective practices within care systems.’ Given innovation at multiple levels, we expect that the proposed study will considerably advance the field in the area of implementation of EBT and offer viable options for improving care for children and adolescents.

### Limitations

The proposed study is one of the first to experimentally examine supervision strategies in community mental health, though it is not without its limitations. First, to obtain similar samples of supervision, some frequently used types of supervision could not be included (i.e., group supervision, ‘drop by’ or informal supervision). Informal supervision, in particular, is likely to be quite frequent, particularly for certain agencies and supervisory relationships. Second, as fidelity is one of our primary outcomes, permission to receive audio recorded TF-CBT sessions is needed, precluding examination of fidelity with the clinician’s full caseload. Third, clinicians invite clients to participate, and despite requests that clinicians invite all who meet eligibility and exclusionary criteria, we cannot be sure that enrolled cases are representative of their caseload. Fourth, conclusions drawn from analyses comparing Phase I and Phase II outcomes may be limited, as child cases could not be randomly assigned to ‘usual care’ supervision or one of the Phase II RCT conditions, given the temporal ordering of the study phases. Fifth, although TF-CBT is similar to other CBT-based approaches, the focus on a specific treatment (i.e., TF-CBT) and treatment population (trauma-exposed) allows for careful examination of the implementation of the protocol’s treatment components with fidelity, but may limit generalizability of findings to supervision of other treatments and populations of focus. Finally, another potential limitation is selection effects; the supervisors who agreed to participate may not be representative of public mental health supervisors in general in that all have received EBT training are positive about TF-CBT as an intervention and the enterprise of implementing EBT in public mental health.

## Conclusions

The proposed study has significance for both implementation science and public health, as the ultimate goal of improving supervision in community-based mental health settings is to increase quality of care and clinical outcomes for children and adolescents through improving clinician fidelity. The timing of the current investigation is particularly opportune, given the current momentum and support for the use of EBTs, as well as increasing attention to accountable care. However, the potential public health impact of wide-scale provision of EBTs cannot be realized when EBTs are not sustained post-initial efforts. Increasingly, federal and state-funded EBT implementation efforts are underway, and in some cases, states and counties mandate use of EBT [86,87]. Given the prevalence and high cost of these initiatives, particularly if ineffective for changing and sustaining clinician practice, it is critical to identify strategies for improving implementation, fidelity, and sustainability, such as EBT-focused supervision, with utilization of strategies that more closely approximate those from fidelity monitoring procedures in research trials.

## Abbreviations

EBT: Evidence-based treatment; RCT: Randomized controlled trial; CBS: Community-based supervisors; DI: Dissemination and implementation; CBT: Cognitive behavioral therapy; BR: Behavioral rehearsal; SM: Symptom monitoring; TF-CBT: Trauma-focused cognitive behavioral therapy; WA TF-CBT Initiative: Washington State Trauma-focused cognitive behavioral therapy initiative; TPOCS: Therapy procedures observational coding system; SPQ: Supervision process questionnaire; PWS: Pre-work survey; SDQ: Strengths and difficulties questionnaire; SWAI: Supervisor working alliance inventory; SAS: Supervision alliance scale; TCU-ORC: Texas christian university organizational readiness for change; EBPAS: Evidence-based practice attitude scale; MBI: Maslach burnout inventory; SCARED: Screen for child anxiety-related emotional disorders; PSC-17: Pediatric symptom checklist-17; BPC: Brief practice checklist; SI: Supervision interview; HGLM: Hierarchical generalized linear models; ICC: Intraclass correlation coefficient.

## Competing interests

The authors declare that they have no competing interests.

## Authors’ contributions

SD is the principal investigator of the proposed study. SD generated the idea and designed the study, was the primary writer of the manuscript, and approved all changes. MP is a Co-I on the study, and he designed and wrote the data analysis plan. ED is a Co-I and in collaboration with SD and others developed the TF-CBT fidelity measure and contributed to the development of other study procedures and measures. LB, SK, and JU are Co-Is on the study and provided input on the study design. KT is the project coordinator and has been involved in finalizing study procedures and implementation. AG and JW are study consultants. All authors were involved in developing and editing of the manuscript and have given final approval of the version to be published.
